# Real-world experience with TTFields in glioma patients with emphasis on therapy usage

**DOI:** 10.3389/fonc.2024.1430793

**Published:** 2025-01-07

**Authors:** Claudius Jelgersma, Joan Alsolivany, Gülsüm Akkas, David Wasilewski, Bastian Gastl, Martin Misch, David Capper, David Kaul, Lars Bullinger, Peter Vajkoczy, Julia Onken

**Affiliations:** ^1^ Department of Neurosurgery, Charité – Universitätsmedizin Berlin, Berlin, Germany; ^2^ German Cancer Consortium (DKTK), Partner Site Berlin, German Cancer Research Center (DKFZ), Heidelberg, Germany; ^3^ Novocure GmbH, München, Germany; ^4^ Department of Neuropathology, Charité – Universitätsmedizin Berlin, Berlin, Germany; ^5^ Department of Radiation Oncology and Radiotherapy Charité – Universitätsmedizin Berlin, Berlin, Germany; ^6^ Radiation Therapy, Health and Medical University Potsdam, Potsdam, Germany; ^7^ Department of Hematology, Oncology and Cancer Immunology, Charité – Universitätsmedizin Berlin, Berlin, Germany; ^8^ Berlin Institute of Health at Charité, Universitätsmedizin Berlin, Berlin, Germany

**Keywords:** tumor treating fields (TTFields), malignant glioma, glioblastoma, compliance, usage, adherence

## Abstract

Tumor Treating Fields (TTFields) has emerged as a significant adjunctive component in the treatment of high-grade gliomas following the EF-14 trial in 2017. The incorporation of TTFields, alongside cyclic temozolomide therapy, has demonstrated improved patient outcomes when the usage exceeds 18 h per day (75% usage). *Post-hoc* analysis of the EF-14 trial has demonstrated that therapy usage exceeding 90% is associated with an additional benefit, while rates above 50% have also proven effective in literature. Given the cost-intensive nature and mild- to- moderate constraints associated with the therapy, our objective is to generate real-world data on therapy usage through a retrospective analysis at a high-throughput academic center. Between June 2015 and February 2022, a total of 113 high-grade glioma patients received TTFields therapy. Eight patients discontinued TTFields therapy within 2 months with less than 50% usage and were excluded from further analysis. For the remaining patients, the median age was 51 years (range: 20–76 years) and the mean preoperative Karnofsky index was 80%–90%. Most of the patients (75.2%) initiated therapy concurrently with first-line treatment, of whom 27.6% started TTFields therapy concomitant to the first cycle of temozolomide. 15.2% started TTFields therapy in the second-line and 9.5% in the third-line setting. The study cohort had an average therapy duration of 9.3 months with 3.2 break days per month. The mean therapy usage was 65.5% (SD 17.6%). Usage was highest during the first 3 months, with rates of 77.7%, 72.3%, and 71.6%, and then dropped to around 60% in the following 6 months. Linear regression found no predictors of usage, such as age, timing of therapy initiation, and duration or gender. 55% of patients continued TTFields beyond the first recurrence. Interestingly, no drop in usage rates was observed before tumor recurrence was communicated. However, after diagnosis, patients exhibited a significant drop in usage to an average of 52.3%. This high-volume, real-world TTFields usage data reveal that the extent of usage falls short of the intended 75%. It highlights the importance of monitoring and promoting adherence to maximize its potential benefits in managing high-grade glioma patients. Furthermore, strategies to expedite therapy initiation and improve long-term adherence are warranted.

## Introduction

Patients diagnosed with WHO grade 4 gliomas are still facing very poor outcomes. Despite all efforts that have been made to prolong survival, the best standard- of- care therapy achieves an average survival of 13.5 months, with a range of 11–20 months (1). In recent years, TTFields have emerged as a therapeutic modality alongside resection, radiation, and chemotherapy. In the first controlled trial (EF-11), TTFields therapy was approved as a feasible treatment without causing severe side effects. This study included 237 patients with recurrent glioblastoma; however, we could not demonstrate the benefits in PFS and OS over best physician choice chemotherapy (2). In a subsequent randomized controlled trial including 695 patients with newly diagnosed glioblastoma, OS extended by 4.9 months (3). According to the study design, TTFields therapy could be continued after tumor progression until second progression or for a maximum of 24 months (3). TTFields are alternating electrical fields of specific frequencies utilized to disrupt rapid cell division and slowing down tumor growth, both *in vivo* and *in vitro* (4). The externally applied electric field exhibits selective toxicity to dividing cells, primarily by interfering with the alignment of the mitotic spindle during cell division, which can lead to cell death. Additional biophysical and biological mechanisms of action have been described, resulting in reduced tumor growth (5). To attain the optimal effect, TTFields therapy usage for a minimum of 18 h per day is recommended and is essential to maximizing the survival benefit. A correlation between usage duration and survival has been demonstrated in previous studies for both primary and recurrent glioblastoma (2, 6, 7). Apart from the therapy not exhibiting severe side effects (8) thus making it highly accessible for patients, it is cost intensive. Additionally, factors such as the required continuous application of TTFields during day and night, frequent hair shaving, and technical errors especially during hot weather conditions may contribute to lower usage (9). So far, there has been no study focusing on a detailed longitudinal cycle-wise usage data analysis and predictors in a large patient cohort. Post-approval studies including the most recent about clinical effectiveness in real-world concentrate more on quality of life, safety, and survival (10–13).

With this study, we provide real-world data on therapy adherence and usage of TTFields in high-grade glioma patients treated at a high-volume academic center in Europe. Usage data demonstrated that the majority of patients fell below the anticipated threshold of 75%. Moreover, at the time tumor recurrence is communicated to the patient, usage rate dropped significantly.

## Methods

The usage data reports were generated and provided by the company Novocure and included cycle-wise usage rates, usage rates during day- and nighttime and therapy break days. One TTFields cycle was defined as 30 days/1 month. A break day was defined as 24 h not using TTFields. Clinical data were collected retrospectively from medical reports and record sheets. This includes patients’ characteristics as age, preoperative Karnofsky index, treatments in the first-, second-, and third lines, and tumor specifications including WHO grading, MRI characteristics, and extent of resection. Survival data were obtained from a resident registration office and updated to May 2023. Analysis was performed with IBM^®^ SPSS^®^ Statistics (Version 26) and GraphPad Prism 9 (Version 9.5.1). Results are given as mean values including standard deviation or median. Student’s t-test or one-way ANOVA was used to compare measurements for different subgroups. Multiple regression models were used to calculate predictors for therapy usage. The significance level (α) for all statistical tests was set at 0.05, indicating that a p-value of less than 0.05 is considered statistically significant.

All analyses involving human subjects were meticulously executed in strict adherence to the principles delineated in the Declaration of Helsinki, with the added assurance of having obtained the requisite ethical approval through the local ethics committee’s deliberation (EA4/154/22).

## Results

### Study population and patient characteristics

**Figure 1:**
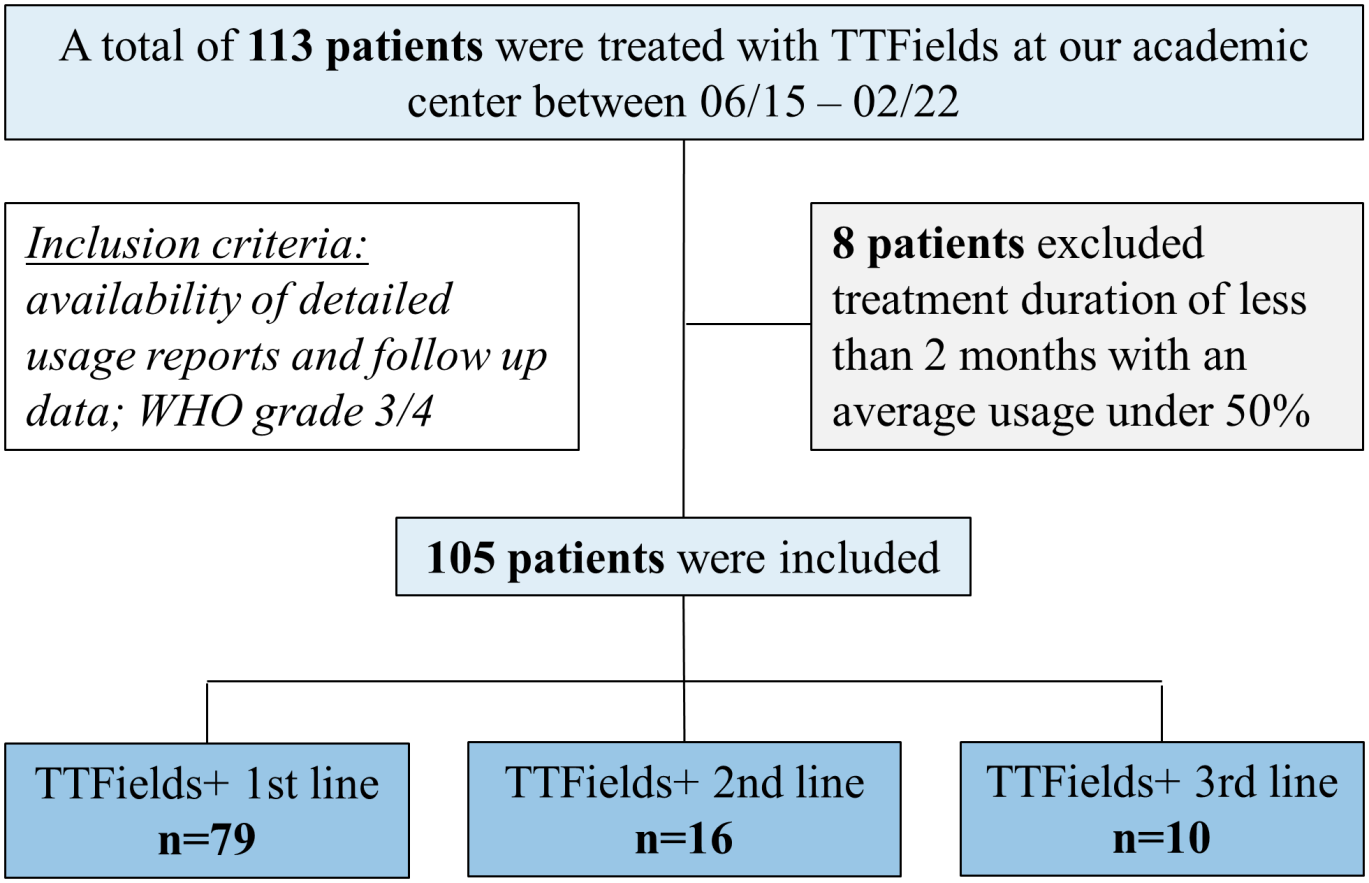
Flowchart of the study population recruitment.

Between June 2015 and February 2022, a total of 113 patients were prescribed TTFields as part of their treatment. Eight patients, who were undertreated with therapy lasting less than 2 months of TTFields and with an average usage below 50%, were subsequently excluded from the analysis. Of the eight excluded patients, three stopped TTFields therapy after one cycle despite demonstrating good average usage. One discontinued due to a severe exacerbation of psoriasis; the reasons for discontinuation for the others were unknown. Thus, a total of 105 patients were analyzed, three quarters (75%) of whom started TTFields together with first-line therapy, 15% with second-line therapy, and 10% with third-line therapy (**Figure 1**).

Patient characteristics are given in **Table 1**. 97.1% of patients were diagnosed with histological grade 4 glioma (WHO 2021). In eight patients, the exact histological grading was not available as they were diagnosed externally. The mean age was 49.6 years (SD 12.8 years; median 51 years, range: 20–76 years), and 62% were men and 38% women. A majority (84%) of patients were in a good clinical condition, with the preoperative Karnofsky index of 80% or above. Details on tumor localization, preoperative MRI characteristics, extent of resection, and first-, second-, or third-line treatments are provided in **Supplementary Table 1**.

**Table 1:** Patients characteristics.

Gender		*N*	*%*	Extension of resection	*N*	*%*
Male		65	61.9	Complete	42	38.9
Female		40	38.1	>90%	28	25.9
				<90%	7	6.5
Age at surgery (y)		*Mean*	*SD*	Biopsy	15	13.9
		49.6	12.8	n.a.	16	14.8
Diagnosis (WHO 2021)	Grade	*N*	*%*	Karnofsky index (%)*	*N*	*%*
Glioblastoma IDH wt	4	87	82.9	100	5	4.8
Astrocyotoma IDH mut	4	7	6.6	90	63	60
Astrocyotoma IDH mut	3	3	2.9	80	20	19
n.a.	4	8	7.6	70	4	3.8
				60	4	3.8
MGMT		*N*	*%*	n.a.	9	8.6
Unmethylated		50	47.6			
Methylated		39	37.1			
n.a.		16	15.2			

N, number of patients; %, percentage of the analyzed cohort; SD, standard deviation; IDH, isocitrate dehydrogenase; wt, wild-type, mut, mutant; n.a., not available; *preoperative.

Of the 87% who received the Stupp regime as first- line therapy, 37% of the patients started TTFields concomitant to the first temozolomide (TMZ) cycle. A further 29% of patients were able to start therapy in the second and third cycles, respectively. For nine patients, the exact start of TTFields remained unknown.

**Figure 2:**
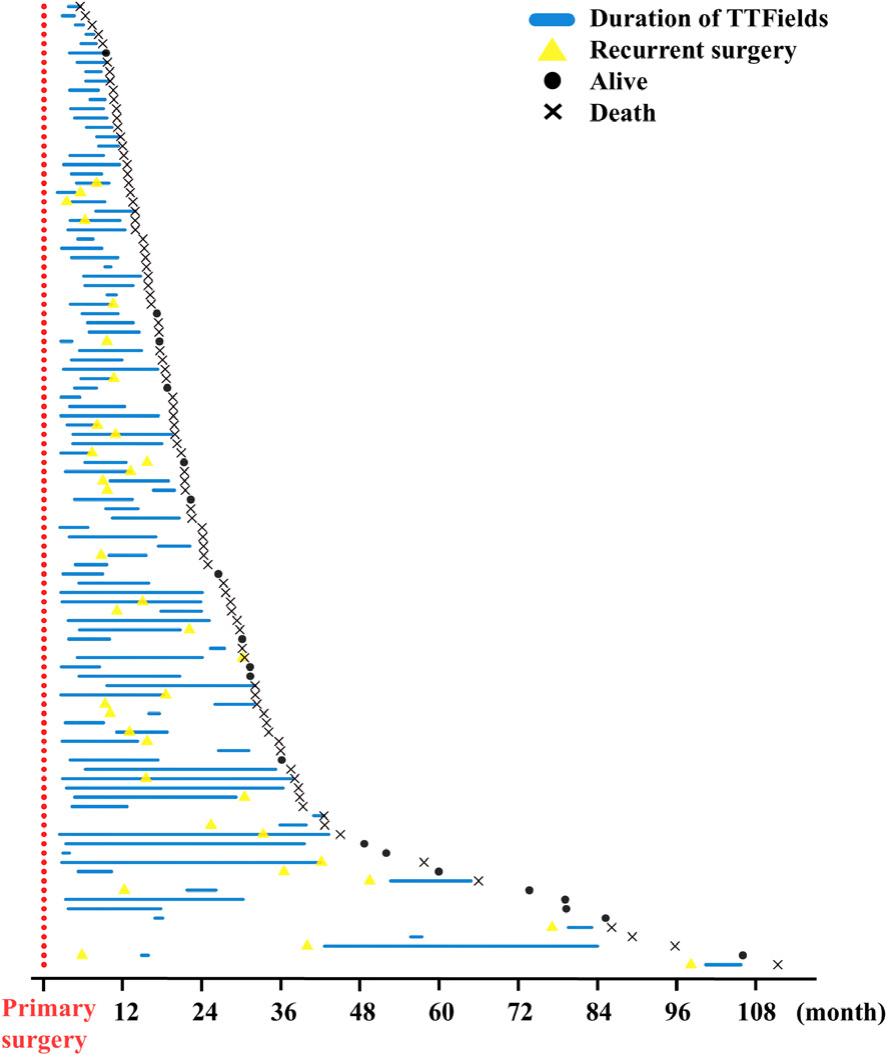
Swimmer plot of TTFields therapy courses in all patients. Primary surgery was defined as day 0 and marked red. Duration of TTFields therapy is shown as a blue line. In case of recurrent surgery, this is indicated by a yellow triangle. Patients’ status at follow- up were marked as a dot if alive and x for death at the end of the study. Patients are not sorted by TTFields start in the first-, second-, or third-line therapy. One patient with follow-up of 4,654 days was excluded to optimize the overview in this figure.

The provided Swimmer Plot in **Figure 2** shows an overview of therapy duration marked by a blue line in relation to the primary surgery, which is for all patients day 0 at the x-axis. Additionally, in case a second surgery was needed, it is given as a yellow triangle. Also, the Swimmer plot provides the patient status at the last follow- up as dead or alive.

### Therapy usage

**Figure 3:**
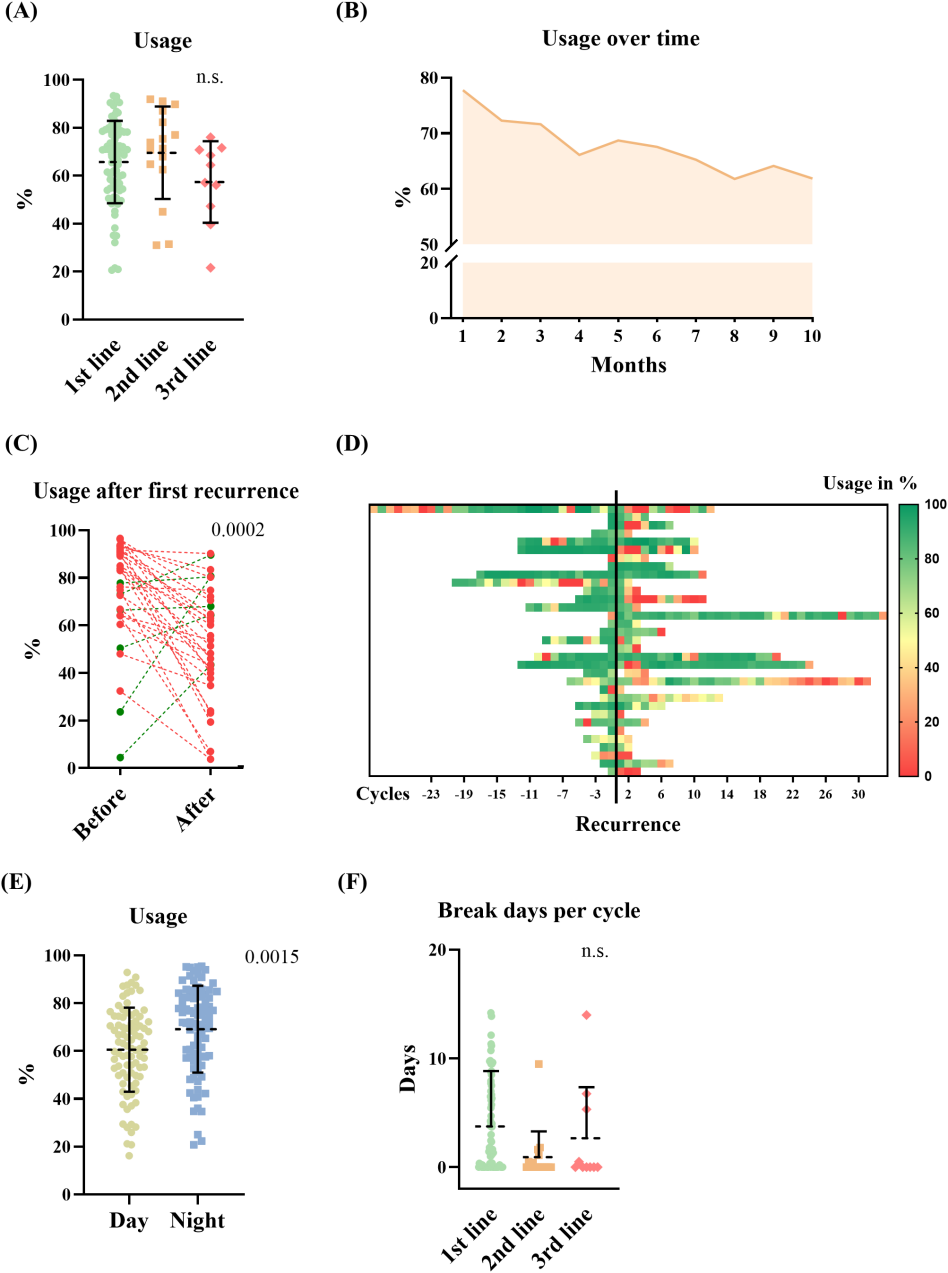
TTFields usage data, and therapy adherence. **(A)** Average usage of TTFields in the first-, second-, and third-line therapy (ANOVA, n.s.). **(B)** TTFields usage in the course of time in all included patients (mean of all patients during each cycle). **(C)** Showing the average (mean) therapy usage of patients before and after recurrence in the first-line therapy when TTFields was continued (red = dropping -, green = increasing usage, Student’s t-test, p = 0.0002). **(D)** Heat map of usage in % per cycle before and after recurrence in the same patients. Yellow to red indicates usage rates below 50%. **(E, F)** Showing usage parameter. **(E)** TTFields usage during day- and nighttime (Student’s t-test, p=0.0015). **(F)** Break days per cycle in different therapy lines (Student’s t-test, n.s.).

The average usage of TTFields therapy for all included patients was 65.5% (SD 17.6%), with a median of 68.9%. We hypothesized that disease stage may have an impact on usage, as tumor progression may be associated with increasing disease burden, neurological deficits, and patient support. Therefore, we investigated how TTFields usage differs in combination with first-, second-, and third-line therapies. The analysis did not show a significant difference between usage in the first-, second-, and third-line therapies (**Figure 3A**; p = n.s.; ANOVA). One- third (32%) of the patients achieved a usage above 75%. 18% did not reach the minimum effective usage of 50%–60% according to Toms et al. (7). Average usages where highest in the first 3 months of TTFields: 77.74%, 72.27%, and 71.61%, respectively. This diminishing trend was observed over the full course of time (**Figure 3B**). Analyzing predictors for extent of usage via linear regression models did not show correlation for age, gender, TTFields start in first-, second-, or third-line therapies, and number of therapy break days (**Table 2**). Also, subgroup analysis of patients who received TTFields in first- line therapy was dichotomized with a threshold of 75% usage, and no predictors were found using the logistic regression model. However, longer therapy duration was associated with higher usage as well as increased break days (**Table 2**). Other predictors influencing the increase of break days were not found (**Table 2**). Subsequently, we examined the correlation between the usage observed in the initial cycle or the first three cycles, and the average usage in the course of time. The analysis revealed a weak correlation with R² coefficients of 0.124 (p=0.000) and 0.175 (p=0.000), respectively.

**Table 2:** Factors evaluated for their impact on usage and therapy break days.

Multiple linear regression (p-values)		
	Usage	Break days
Gender	0.14	0.083
TTFields in 1st, 2nd, 3rd line	0.794	0.297
Age	0.165	0.318
Therapy duration	0.003	0.000
Break days	0.105	–
Average usage	–	0.105

Next, we analyzed therapy usage in patients who experienced recurrence while receiving TTFields in the first-line therapy. Recurrence was documented in 76%. Of these, TTFields therapy was continued in 55%. The average usage before the first documented progress was 74.2% (SD 22.4%) and decreased significantly (p=0.0002, Student’s t-test) to 52.3% (SD 24.1%) after the progress was communicated to the patient as shown in **Figures 3C, D**. The survival data for patients who started TTFields therapy in their first-line treatment were as follows: the mean overall survival was 24.8 months (SD 16.1 months; median 20 months), and the mean progression-free survival was 15.8 months (SD 13.7 months; median 12 months).

### TTFields adherence

To assess therapy adherence, break days and usage routine during day and night were analyzed. We found that TTFields was significantly more utilized during the night compared with average daytime usage (**Figure 3E**, p=0.0015, Student’s t-test). TTFields were administered with a mean of 9.3 months SD (9.3 months; median 5,9 months) on average. Patients who initiated TTFields concurrent with their first-line treatment had a mean duration of 10.2 months on TTFields (SD = 9.6 months). Starting TTFields therapy in the context of second-line treatment, the mean duration on TTFields was 8.5 months (SD = 9.6 months), while for the third-line treatment setting, it was a mean of 3.2 months (SD = 1.4 months). Statistically in one-way ANOVA, no significance was shown (p=0.071). The mean number of therapy break days per TTFields cycle was calculated with average 3.2 days (SD 4.8 days), not showing a significant difference between therapy lines (first line: 3.8 days/TTFields cycle, SD 5.1 days; second line: 0.9 days/TTFields cycle, SD 2.4 days; third line: 2.7 days/TTFields cycle, SD 4.7 days; n.s. in one-way ANOVA) (**Figure 3F**).

## Discussion

The principal novel finding of the study is that average TTFields therapy usage in this large real- world cohort of glioma patients falls below the intended 75% margin. Nevertheless, two- thirds (67%) of patients managed to achieve an average usage of more than 60%. Furthermore, this study could not identify factors influencing therapy usage. A robust usage observed within the initial 3 months may serve as a surrogate indicator for an effective usage rate in the subsequent months. Interestingly, recurrences were not predicted by a decline in usage as it could have happened due to neurological decline from tumor progression; rather, a significant drop occurred following the communication of the recurrence to the patient.

To our knowledge, this study has the largest cohort size published concerning the analysis of real-world usage data of TTFields (14). We included 105 patients receiving TTFields during the course of disease. Based on the low dropout rate of 8 out of 113 patients, a good pre-selection of patients by prescribing physicians can be assumed. Even though the intended treatment start of TTFields was concomitant to the first TMZ cycle according to the EF-14 trial protocol (6), only one-third of our total cohort obtained TTFields in the first cycle. 65% of patients managed to start within the first three TMZ cycles, which indicates the need for infrastructure improvements to expedite patients’ access to therapy.

The effectiveness of a structured consulting strategy to increase TTFields therapy acceptance has already been evaluated by Proescholdt et al. (15). They reported that the acceptance rate increased to 68% and that the average usage rate exceeded 90% when patients received an additional counseling appointment about TTFields, analogous to consultation of radiation therapy and chemotherapy (15). Ensuring early dissemination of information regarding TTFields and expediting the administrative prescription process may also prove advantageous in ensuring a prompt commencement of therapy. Treatment consultations and prescriptions for this cohort were provided by treating neurosurgeons, oncologists, and radiotherapists without a standardized structure. This has improved over the years as awareness of its importance has increased. Nevertheless, the implementation of standardized and systematically scheduled reevaluations should become routine outside of prospective clinical studies.

We observed a broad range of therapy usage in our cohort. Notably, there was no significant difference in average usage whether patients started TTFields in the first-, second-, or third-line therapy. In total, the average usage of the entire cohort was 65.5% (mean), falling below the threshold of 75% as defined in the EF-14 trial (6). Nevertheless, approximately two-thirds of the patients still exhibited a therapy usage exceeding 60%, which has been associated with a statistically significant survival benefit (7). In our study, 15% of patients had an average usage rate between 50% and 60%, which is the minimum threshold at which Toms et al. observed a survival benefit (7). However, 18% of patients included in our study, fell below this minimum threshold. Interestingly, smaller prior single-center analyses have reported a variety of average usage rates between 60% and 91.6% depending on centers (11, 16–19). Notably, the first study conducted in our clinic, encompassing a cohort of 40 patients undergoing TTFields in both primary and recurrent GBMs, reported higher mean usage rates of 86% (9). The considerable discrepancy could potentially be attributed to a more stringent patient selection in the beginning of the TTFields era by the prescribing physicians.

Previous studies have shown that the usage is correlated directly with overall and progression-free survival (7). Ballo et al. showed that average usage in the first 3 months was significantly associated with overall survival (16). Our descriptive individualized analysis revealed that patients with usage below 50% showed a rapid decline in the earliest therapy months (**Supplementary Figure 1**; showing the individual course of usage in patients with average usage below 50%). Vice versa, we identified a weak positive correlation between the usage within the initial (first or first three) months and the usage over time. Similar to Ballo et al. (2022) (16), patient- and tumor-specific factors, particularly tumor progression, could not be identified as influential variables on usage. However, a statistically significant threshold could not be established. Another consideration was whether clinical deterioration due to a recurrent tumor could lead to a decline in usage. Conversely, whether a drop in usage could indicate an impending tumor recurrence. We were able to observe a significant drop in usage to a mean of 52.3% shortly after tumor recurrence was communicated to the patient. Therefore, the average usage fell into the lowest usage subgroup of 50%–60% for which a statistically significant survival benefit of 2 months has been reported previously (7). Given that the TTFields usage only slightly exceeds the lowest reported efficacy threshold, a critical perspective is warranted in terms of cost-effectiveness balance for these patients (7, 10, 20).

This highlights the need for additional supportive interventions and more rigorous follow-up of our patients to maintain effective treatment thresholds. Looking forward, the prospective, multicenter, randomized trial COCOON (NCT06017063) holds promise to provide further insight whether additional structured psycho-oncological video interventions for patients and caretakers can improve patients’ TTFields therapy usage rates.

## Limitations

Our intention was to analyze TTFields therapy usage data within a real-world setting. It is crucial to acknowledge additional limitations, including the single-center design, retrospective nature, and the inclusion of patients with varying disease stages at the time of TTFields therapy prescription, as well as different tumor genetics. Owing to the retrospective design, the reasons for low usage rates were not available. To facilitate a more robust and comprehensive analysis, a prospective observational study, preferably in a multicentric approach, would be necessary to overcome these limitations. Additionally, the aspect of quality of life impairment over time should be considered, which is absent in the present study due to its retrospective design.

## Conclusion

In summary, the study shows that TTFields usage falls below the aimed 75% margin in the real-world setting in our center, particularly after the initial 3 months of treatment as well as after communication of tumor recurrence to the patients. These findings highlight the importance of monitoring and promoting adherence to TTFields therapy to maximize its potential benefits in the management of high-grade gliomas. Furthermore, strategies are warranted to expedite the start of therapy and improve long-term adherence.

## Data Availability

The raw data supporting the conclusions of this article will be made available by the authors, without undue reservation.
