# Supplementary material for: Real-world experience with TTFields in glioma patients with emphasis on therapy usage
Source: Front Oncol. 2025 Jan 7;14:1430793. doi: 10.3389/fonc.2024.1430793 (PMC11747310; doi:10.3389/fonc.2024.1430793)
Supplement: Supplementary file 1 [file DataSheet1.pdf]

**Primary tumor localization**		*N*	*%*
	**left**	36	34
	**right**	60	57
	**both**	9	9
	**Frontal**	43	41
	**Temporal**	45	42,9
	**Parietal**	34	32,4
	**Occipital**	12	11,4
	**Infratentoriell**	5	4,8
	**Eloquent**	46	43,8
	**Multilocal**	22	21
**Contrast enhancement**		*N*	*%*
	**no**	10	9,5
	**yes**	84	80
	**n.a**	11	10,5
		Mean	*SD*
	**FLAIR volume (cm³)**	106,4	159,1
	**Contrast volume (cm³)**	24,5	26,7
	**n.a**	23	22
**1st progress**		N	%
		86	81,9
	Local	57	54,3
	Distant	19	18,1
	Boths	4	3,8
	n.a.	6	5,7
**2nd progress**		56	53,3

**1st line**		N	%
	**Stupp**	91	86,7
	**RTx mono**	3	2,9
	**TMZ mono**	2	1,9
	**Nordic**	2	1,9
	**CeTeg**	1	1
	**Stupp + CCNU**	1	1
	**RTx + CeTeg**	1	1
	**Best supportive care**	1	1
	**Not assessed/not available**	3	2,9
	total	*105*	*100*
**2nd line**	**Metronomic TMZ**	33	31,4
	**Stupp**	11	10,5
	**RTx + metronomic TMZ**	8	7,6
	**RTx mono**	6	5,7
	**TMZ mono**	3	2,9
	**RTx + CCNU**	3	2,9
	**others**	2	1,9
	**PCV**	2	1,9
	**CeTeg**	1	1
	**Bevacizumab**	1	1
	**CCNU**	1	1
	**PCV**	1	1
	**Best supportive care**	7	6,7
	**Not assessed/not available**	4	3,8
	total	*83*	*79*
**3rd line**	**Metronomic TMZ**	9	8,6
	**CCNU**	9	8,6
	**Regorafenib**	4	3,8
	**RTx mono**	3	2,9
	**Bevacizumab**	3	2,9
	**RTx + metronomic TMZ**	3	2,9
	**RTx + CCNU**	3	2,9
	**Bevacizumab + CCNU**	2	1,9
	**PCV**	2	1,9
	**CeTeg**	1	1
	**PCV**	1	1
	**Bevacizumab + metronomic TMZ**	1	1
	**Best supportive care**	7	6,7
	**Not assessed/not available**	8	7,6
	total	*56*	*53,3*

SUPPLEMENTARY TABLE 1: left site: Descriptive MRI data of primary tumor localization, characteristics of contrast enhancement as well as rates of 1st and 2nd progression. Right site: detailed therapy regimes of 1st, 2nd and 3rd line therapy are shown.

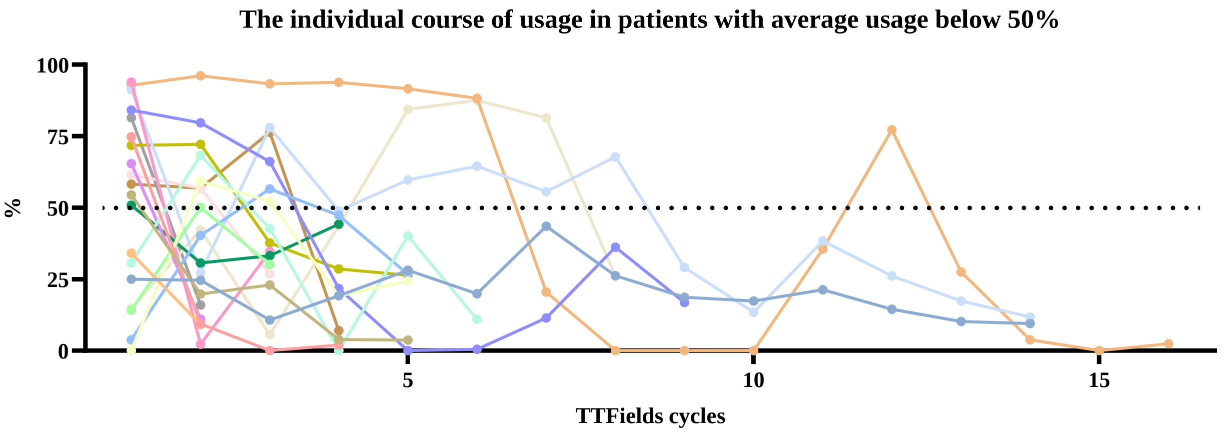

SUPPLEMENTARY FIGURE 1: Overview of patients with average usage below 50% and their detailed course of usage over time
